# Allele Workbench: Transcriptome Pipeline and Interactive Graphics for Allele-Specific Expression

**DOI:** 10.1371/journal.pone.0115740

**Published:** 2014-12-26

**Authors:** Carol A. Soderlund, William M. Nelson, Stephen A. Goff

**Affiliations:** 1 BIO5 Institute, University of Arizona, Tucson, Arizona, United States of America; 2 iPlant Collaborative, University of Arizona, Tucson, Arizona, United States of America; University of North Carolina at Charlotte, United States of America

## Abstract

Sequencing the transcriptome can answer various questions such as determining the transcripts expressed in a given species for a specific tissue or condition, evaluating differential expression, discovering variants, and evaluating allele-specific expression. Differential expression evaluates the expression differences between different strains, tissues, and conditions. Allele-specific expression evaluates expression differences between parental alleles. Both differential expression and allele-specific expression have been studied for heterosis (hybrid vigor), where the hybrid has improved performance over the parents for one or more traits. The Allele Workbench software was developed for a heterosis study that evaluated allele-specific expression for a mouse F1 hybrid using libraries from multiple tissues with biological replicates. This software has been made into a distributable package, which includes a pipeline, a Java interface to build the database, and a Java interface for query and display of the results. The required input is a reference genome, annotation file, and one or more RNA-Seq libraries with optional replicates. It evaluates allelic imbalance at the SNP and transcript level and flags transcripts with significant opposite directional allele-specific expression. The Java interface allows the user to view data from libraries, replicates, genes, transcripts, exons, and variants, including queries on allele imbalance for selected libraries. To determine the impact of allele-specific SNPs on protein folding, variants are annotated with their effect (e.g., missense), and the parental protein sequences may be exported for protein folding analysis. The Allele Workbench processing results in transcript files and read counts that can be used as input to the previously published Transcriptome Computational Workbench, which has a new algorithm for determining a trimmed set of gene ontology terms. The software with demo files is available from https://code.google.com/p/allele-workbench. Additionally, all software is ready for immediate use from an Atmosphere Virtual Machine Image available from the iPlant Collaborative (www.iplantcollaborative.org).

## Introduction

Sequencing the transcriptome can answer various questions such as determining the transcripts expressed in a given species for a specific tissue or under a certain condition, evaluating differential expression (DE), discovering variants, and evaluating allele-specific expression (ASE). DE studies analyze the expression similarities and differences between species, tissues and conditions. ASE studies analyze the difference in expression between the parental alleles, where if one allele has significantly greater expression, it is referred to as allele imbalance (AI). Both ASE and DE require that the reads be trimmed and aligned to the reference genome. ASE further requires variant calling, calculation of SNP and/or transcript read coverage, and a test for AI. DE further requires the number of reads aligned to each transcript and a test for significance. This manuscript is mainly focused on allele imbalance, however, the data can also be used for DE analysis, and is briefly discussed.

When mapping reads to a reference genome, there can be significant bias against reads with alternative alleles. One solution has been to mask the alleles, but as shown by Degner et al.[1], this can still lead to a 5–10% bias. GSNAP [2] presents an alternative solution by allowing SNP-tolerant mapping, which takes into account all combinations of major and minor alleles, however, their README file [3] states “with longer reads now of 75 or more bp, GSNAP alignments are generally fine without SNP-tolerant alignment”. Satya et al. [4] provide software for building an enhanced genome that contains every possible haplotype of a length r segment (where r is a fixed read length). Stevenson et al. [5] proposed limiting the number of mismatches to the number of differentiating sites for the region. AlleleSeq [6] creates and aligns to the maternal and paternal genome. MMSeq [7] aligns to a sample-specific transcriptome constructed with phased or un-phased parental genotypes. Quinn et al. [8] discuss why variant-calling is more accurate using genome data than RNA-Seq data, then present an ASE protocol that uses RNA-Seq data and creates an alternative reference after filtering SNPs not found in a high-quality SNP database.

SNP discovery is important for problems ranging from human disease studies to species comparisons. Over 20 variant callers have been published in the recent years. Many of them concentrate on the problems of finding rare variants in pooled samples, genotype calling, tumor-normal pairs, exome studies, and low coverage. These problems are found in human disease studies, i.e. most variant software is written for human disease studies, though most have been used for other SNP discovery challenges. The variant callers may incorporate quality, alignment, experimental errors, and dbSNP alleles. Most of them use Bayesian statistics. Yu and Sun [9] compared four popular variant callers (SOAPsnp [10], SAMtools [11], GATK [12], and Atlas-SNP2 [13]) and found low agreement between the programs when focusing on between 3x and 10x coverage, though agreement was good with high coverage and quality. It is now customary to read as input the BAM alignment files and output a VCF (variant call format) file[14], which can include the allele genotype counts.

ASE has been studied for imprinting and cis-regulation (reviewed in [15]) and for heterosis [16]–[18]. ASE software has been written for humans and F1 hybrids. For human ASE studies, there is only one genome involved but two parental haplotypes, where the genotypes need to be phased to obtain the correct maternal and paternal genome. For F1 studies, the parents are genetically distinct. The parents may be inbreds (homozygous) where ideally one of them is the reference strain, in which case, the alternative allele of a hybrid SNP automatically belongs to the alternative parent. If the parents are heterozygous and there is no SNP file representing the alleles of the parents, then the parental alleles cannot be assigned unambiguously with only a reference sequence and hybrid RNA-Seq data. However, if the transcriptomes of the parents are also sequenced, then the RNA-Seq alignment may be used to determine if the parents are homozygous for hybrid SNPs, and hence assign the parental allele. There are four published distributed packages for ASE computation, where ASARP [19] and AlleleSeq [6] are specifically written for human genomics. Allim [20] is the only pipeline written for F1 individuals and takes various types of input to unambiguously assign parental alleles. It uses GSNAP to map reads to the genome, SNPs are identified and used to create a polymorphism-aware genome, and GSNAP is used again to map the reads against it. MMSeq [7] works with diploid organisms where phasing is optional; it creates heterozygous transcripts, maps the reads to the transcripts, and simultaneously computes transcript-level expression and allele imbalance at the transcript level. There are other transcriptome pipelines, such as ArrayExpressHTS [21], which perform many of the same steps, but without taking into account reference bias or computing ASE.

Allele imbalance has been computed using a variety of statistical tests, such as chi-square [18], [19], binomial test [1], [6], [22], Bayesian model [23], [24] and g-test without replicates and ANOVA with replicates [20]. ASARP [19] show that a minimum of 20 reads is required to get adequate statistical power, which is the typical cutoff used by most studies. AI may be computed for SNPs, exons, transcripts, or genes. If AI is computed for the transcript, then it is typical to sum the heterozygous SNP coverage to represent heterozygous transcripts; if a read covers multiple SNPs, it should only be counted once. A consideration when summing the SNP coverage is that there may be SNPs that have the opposite direction from the majority. In a related problem, Skelly et al. [23] address variable ASE by labeling such genes, where variable ASE is due to alternative splicing, start site or poly-adenylation. To obtain the full heterozygous read count, MMSeq [7], Cufflinks [25], and eXpress [26] compute it by assigning each read containing a SNP to the (putatively) correct parental transcript isoform along with assigning all non-SNP reads according to the ratios inferred from the SNP-containing reads.

To our knowledge, there is no freely available graphical interactive package for exploring allele imbalance data. This is complex data that may contain one or more libraries, one or more replicates per library, unique and shared exons between transcripts, variants with their computed effects, and allele imbalance for SNPs, transcripts and/or genes. Though pipelines can produce the ‘list of allelic imbalance” genes, transcripts or SNPs, that does not allow the scientist to explore the data for complex relations and novel discovery. This is a tremendous amount of data and processing steps, and there are many places where errors can arise and genomic data typically has measurement error; it is extremely hard to detect insidious problems when viewing flat files of information. Therefore, next generation sequencing results should be stored in a database with a query interface. Having the data in a queryable database provides a much more organized methodology for subsequent research involving the data and results, and allows scientists to gain a better understanding of their data. Moreover, when using a language such as Java that can be executed on the web, the data and results can be easily shared with the scientific community.

For a heterosis study, we sequenced the transcriptome of two inbred mice and their hybrid progeny using four different tissue types in order to study the hypothesis that the hybrid can discriminate between alleles encoding proteins that do not fold efficiently or are unstable for a substantial saving of metabolic energy during folding of the protein [27]. To test this hypothesis, we wanted to discover transcripts with allelic imbalance to determine if the higher expressed allele folds correctly and the lower expressed allele does not. We also wanted to study the differential expression between the inbreds and the hybrid. This study necessitated installing numerous software programs, and developing our own database with an interactive Java interface (referred to as Allele Workbench (AW)) to explore this wealth of information. The software has been engineered such that it can be used by other projects, is fully documented, and includes a demo dataset.

This manuscript presents the AW pipeline, interface to build the AW database, and the AW interactive graphics program. The AW pipeline is specific to F1 hybrid processing with inbred parents, but the database and interactive graphics can be used for any ASE data. As discussed above, there are a range of programs that can be used for each step of the pipeline; our pipeline provides a default set along with the necessary workflow, and each step can be replaced with an alternative program with the condition that the output be provided as specified by the pipeline. The AW reduces allele bias by creating a variant masked genome and aligning with Bowtie2 [28] via Tophat2 [29], which allows a reduced penalty for masked bases. The AW provides both heterozygous SNP coverage and isoform-aware read counts. It uses the binomial test to determine the allele imbalance for the genes, transcripts and SNPs, where the test is applied to both SNP coverage and read counts for the genes and transcripts. Transcripts are labeled that have significantly opposite-directional SNP imbalance or replicate values. The interactive graphics allows the user to query the AI by SNP or transcript. To study the folding of proteins encoded by AI transcripts, it computes SNP effects and the parental protein sequences. This manuscript also briefly discusses the Transcriptome Computational Workbench [30], as it can be used with the output of the AW pipeline to study the differential expression of the same libraries that are analyzed for ASE in the AW interactive display. Both the AW and TCW are written in Java, use MySQL databases, and can be executed either stand-alone or as a web applet. Results from our full mouse study will be published elsewhere, but the mouse data is used here to demonstrate the utility of the software and provide some initial results. The Discussion section covers three published studies and how they could have used the AW and TCW to make their processing more efficient.

## Materials and Methods

The naming of the raw files is very important (described in the AW documentation), as the name will be retained through each step of the pipeline, and then used in the AW program to build the database, which maps each file to its respective library conditions, e.g. strain and tissue. The AW pipeline steps and Java processing are listed in Table 1.

**Table 1:** Steps of the AW pipeline and Java processing.

Program description	Input^1^	Output^1^^,^ 2	Tool used; Postprocessing
QC^3^	Raw read files	HTML files with quality measures	FastQC [31]; Merge to single HTML file
Trim^3^	Raw read files	Trimmed read files	Trimmomatic [32]
Align to GS^3^^,^ 4	Trimmed read files, GS files, annotation file	Alignment files	Tophat2 [29], Samtools [11]
Variants^3^^,^ 4	Alignment files	Variant files	Samtools, bcftools [11]; Merge sample results into the consensus VCF
Mask GS^3^	GS, variant files	Masked GS	Bedtools [33]
Align to masked GS^3^	Trimmed read files, masked GS, annotation file	Alignment files	Tophat2, Samtools
SNP coverage^3^	Alignment files, variant file	SNP coverage files	Samtools; Parse counts from mpileup output
Transcripts counts^3^	Parental transcript files^5^	Heterozygous count files^5^, total count files	STAR [34], eXpress [26]; Create total count files
AW build database (runAW)^6^	SNP coverage files, annotation file, variant file; Optional: GS files, heterozygous count files^5^, NCBI annotation	AW database, parental protein files, parental transcript files^5^	-
TCW build database^6^	Transcript or protein file; total count files	TCW database	BLAST [44], edgeR [39], GOseq [42]

1 Raw read files (.fastq), GS (genome sequence, fasta), annotation file (.gtf), alignment file (.bam), variant file (.vcf), SNP coverage (.bed), transcript counts (.xprs).

2 Though not listed in their output column, all scripts output an.html summary file. The two Java build programs enter summary information into their database for display by their Java query program.

3 Pipeline scripts are Perl, except QC is shell. Each script executes one or more tools on all input files, renames the result files with their library abbreviations, puts them into the/Results directory, and writes the summary.html file.

4 These steps are only necessary if the variant file is not available.

5 runAW must be executed before the “Transcripts counts” step to produce the parental transcript files and again afterwards to update the database with the transcripts heterozygous count files. The optional AW build files are not needed for the initial build.

6 Java graphical interface.

### AW Pipeline

The Allele Workbench comes with a number of scripts and third-party tools organized to facilitate batch processing of NGS data for DE and ASE studies. These fall into four categories:

1. Sequence preparation
2. Variant calling, if necessary
3. Alignment to reference genome and extraction of heterozygous SNP coverage
4. Alignment to transcripts and quantification of heterozygous and total transcript read counts

The batch scripts are designed in a modular fashion so that the user can easily substitute steps with an alternative method, and verify the results of each step before continuing. The exact steps will vary somewhat depending on data, as described below. Each script takes as its primary input a directory of files, calls one or more underlying tools to process these files, performs any necessary post-processing, and outputs to a standardized directory named "Results". Most of the scripts generate an overall summary, which should be checked before going to the next pipeline step. For tools that are not natively threaded, the scripts implement multiprocessor operation by splitting the set of input files. All necessary tools are supplied with the package (or local installations may be used). Table 1 describes the steps of the pipeline, and the following provides a brief description of each.

#### 1. Sequence Preparation

NGS reads should be trimmed to remove barcodes, adapters, and/or low quality sequence from the beginning and end of the reads. Unless these sections are identified and trimmed off, the majority of reads will not align properly. To identify regions needing trimming, the AW provides a batch script *QC.sh*. The script calls the underlying tool FastQC [31], which generates HTML reports and images showing quality metrics; the script then collects the individual reports into one large HTML output showing all samples. The most important metrics are Per-Base Sequence Quality, which reveals how much leading/trailing trimming is needed to get to good quality sequence, and Overrepresented sequences, which can show if adaptors or other protocol remnants are present on the ends.

To perform the trimming, a batch script *trim.pl* is provided, which runs Trimmomatic [32]. Trimmomatic has a number of trimming modes, which are described in its documentation; all modes are available through the batch script. The batch script also checks for pairing of the files, if they are specified as paired-end. After trimming, the quality should be re-verified using *QC.sh*, and additional trimming performed if necessary.

#### 2. Variant calling

If variants are not already known for the species, then they must be called using the samples. The RNA-Seq reads must first be aligned to the reference genome using the *Align.pl* script, preferably using alternative inbred reads, if available. For variant calling, the batch script *Variants.pl* runs the samtools caller on each sample alignment file (.bam), generating one call set (.vcf file) per alignment, and then combines all of these into a single consensus call set using a user-adjustable threshold (by default, called in at least 5 of samples; the expected genotype can also be set, e.g. 11 for calling on inbred alternate reads, or 01 for hybrid reads).

The alignment files from this step are not suitable for ASE computation because they were not built from a masked genome, hence will suffer from reference bias.

#### 3. Alignment to reference genome and extraction of heterozygous SNP coverage

As mentioned in the Introduction, mapping two species to a reference genome will be biased toward the reference. Therefore, the SNPs should be masked before aligning the hybrid RNA-Seq reads to reduce reference bias, which is performed by the *GSmask.pl* script (using Bedtools [33]).

The trimmed read files are aligned to the reference using the batch script *Align.pl*, which calls Tophat2 to perform the alignment. Tophat2 uses Bowtie2 [28] which has a new parameter (—np, not available in Bowtie1) to set a lower penalty for ambiguous characters, allowing more reads to be mapped. The script *snpASE.pl* then calculates the heterozygous coverage counts for each SNP location by calling "samtools mpileup" and parsing the output. Using runAW, the SNP heterozygous counts are loaded into the AW database; indels are loaded into the AW database for informational purposes but their heterozygous counts are not computed.

#### 4. Alignment to transcripts and quantification of heterozygous and total transcript expression

The script *transASE.pl* computes read counts using the programs STAR [34] and eXpress [26]. Before running this step, a set of parental transcripts must be generated, where the alternative transcripts are obtained by substituting the known SNPs and indels into the reference transcript sequences. As described in the next section, these transcripts sets are generated by runAW once the initial database has been created with the genome reference, annotation, and variants.

The trimmed read files must then be aligned to the parental transcript set. It is essential in this alignment to allow multi-mapping since the transcripts will include splice variants having many shared segments; moreover, the eXpress documentation recommends setting very relaxed multi-mapping parameters and allowing eXpress to determine the correct assignment weights using its statistical models. Bowtie2 is not well-suited for this purpose since it runs very slowly with multi-mapping options enabled (-k, -a); for this reason the alternate aligner STAR has been included in the package, and the batch script *transASE.pl* runs both STAR and eXpress in succession to generate the transcript expression levels.

The eXpress-generated count files (extension ".xprs") are placed into the "Results" directory, and may be loaded into the AW database using runAW. A directory "ResultsTCW" contains the total counts, i.e. the transcript counts summed over both alleles and counts where there are no variants. These read count files may be loaded into TCW for DE analysis between samples.

### Build the AW database

**Figure 1:**
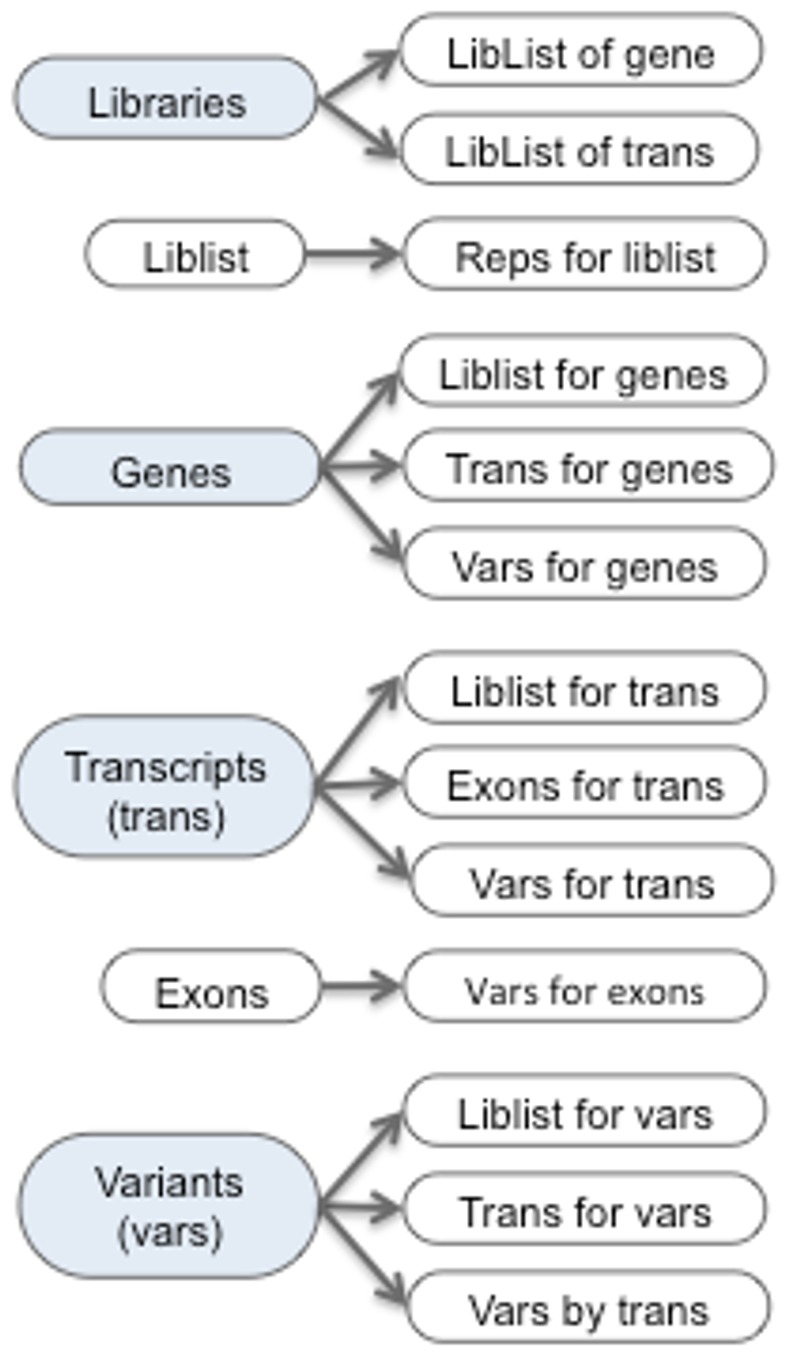
viewAW tables. The blue circles represent tables that can be queried in viewAW. From each table, one or more rows may be selected to view the associated table of data, which is indicated by the pointed-to circles. The “LibList” is the library counts for a selected set of genes, transcripts or SNPs, which link to the associated replicate counts.

The runAW graphical interface builds the AW database using no external programs. The required input is the genome annotation file, VCF file, genome sequences files, and SNP coverage.bed files. Optional input is the NCBI gene annotation file, a variant effect file, and the eXpress transcript count files. The user defines the library conditions and abbreviations, which are used to map the SNP coverage and transcript read count files to their respective libraries. The runAW program is first run to build the database and provide the computations discussed below, but can then be run again to add or update any of the optional files. The initial build populates the libraries, gene, transcripts, exons and variant tables, and provides the relations between these entities as shown in Fig. 1. An overview summary is computed, where many of the results in the summary are presented in the Results Section.

The AW represents read coverage per SNP, summed SNP coverage per transcript and gene, read counts per transcript, and summed transcript read counts per gene. SNP coverage counts are summed to approximate transcript and gene allele-specific expression. The corresponding p-value has the caveat that counts for nearby SNPs are not fully independent; AW mitigates this problem by only counting one representative SNP from each cluster of closely spaced SNPs. For the summed SNP coverage, all transcripts that contain a SNP include its count. In contrast, the read count computation only assigns any given read to one transcript taking into account variants and alternative isoforms. The transcript with the highest read count represents the most likely expressed transcript.

Both SNP and read counts are performed per replicate, which are summed for a total count. Each gene is assigned a start coordinate of its leftmost 5′ transcript start and an end coordinate of its rightmost 3′ transcript end, includes all SNPs within that range, and uses the summed read counts from its transcripts. The p-values for each SNP, transcript, and gene are calculated using the respective heterozygous counts and applying the binomial test. SNPs must have heterozygous counts ≥20, and genes and transcripts must have at least one SNP with heterozygous counts mg20, otherwise the p-value is marked undefined. For genes and transcripts, the heterozygous read counts must be ≥20, otherwise the p-value is undefined. The transcripts for a gene are ranked according to the number of reads, where rank = 1 is the most likely transcribed isoform.

SNPs and transcripts are flagged if at least one replicate has a significantly opposite direction (chi-square p-value <0.05) from their summed value. Transcripts are flagged if, for any library, they have at least two AI (p-value<0.05) SNPs in different directions.

The genome sequence, GTF annotation and VCF variants are used to create files of the parental transcripts and protein sequences. If variant effect annotations are not input from an external program, the runAW computes basic variant effects (i.e. 5′UTR, 3′UTR, missense, non-synonymous). It does not assign the effect terms to the same detailed level as snpEFF [35] or Ensembl Variant Effect Predictor (EVP) [36], so these files are optional input to the runAW program. Depending on the genome, the EVP file may include SIFT [37] information, which the AW marks as ‘damaging’ SNPs. The snpEFF file tags some variants as ‘high’ (e.g. frameshifts), which are marked as damaging in the AW. The EVP and snpEFF are not part of the AW package, but EVP is executed from the web, and the snpEFF is downloadable and easily executed, especially if using one of their>2500 known genomes.

### AW query and display

**Figure 2:**
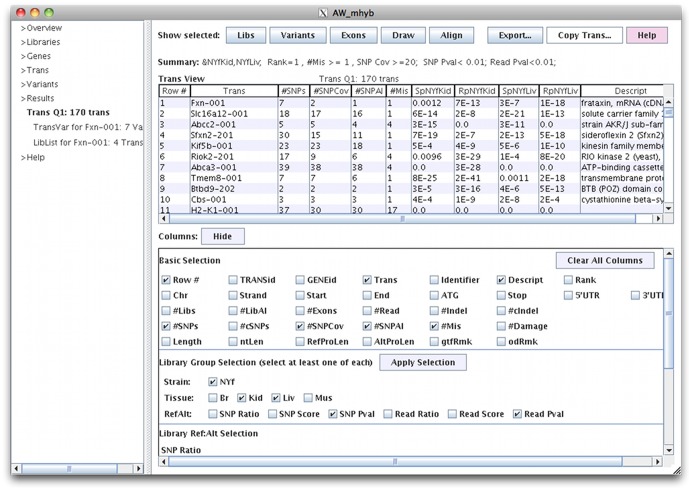
viewAW transcript table. The columns are shown in the lower panel; when an adjoining box is checked, the corresponding column is shown in the table. Selecting “Hide” closes the column listing. The SpNYfKid and SpNYfLiv columns are the SNP coverage p-values. The RpNYfKid and RpNYfLiv are the read counts p-values. The #SNPCov is the number of SNPs with ≥20 reads for any library, #SNPAI is number of SNP that are AI (p-value <0.05) for any library, and #Mis is the number of missense SNPs. #SNPCov and #SNPAI take into account all four libraries, where only two are shown but the others can be viewed by selecting their respective column box next to “Tissue”.

**Figure 3:**
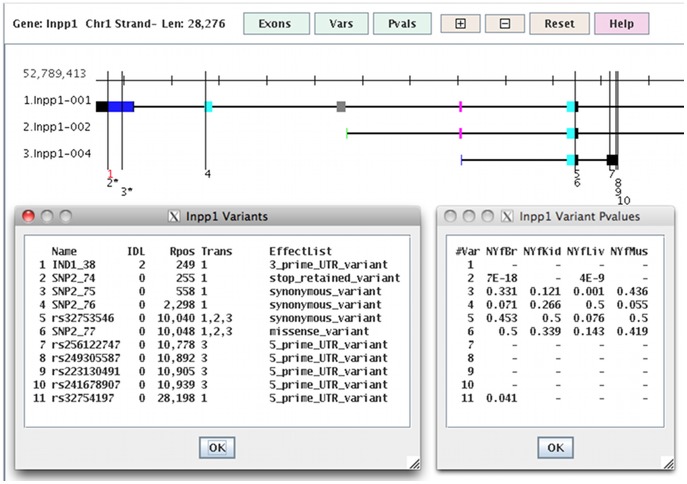
viewAW drawing of a gene with three transcripts and 11 variants. The black exons are non-coding. The coding exons that are stacked but are different colors have different coordinates, e.g. the stack with two pink exons (the same) and a blue (different). The long vertical lines represent SNPs (black) and indels (red); if the number below the variant line is followed by an “*”, then it is AI (p-value <0.05) for at least one library, e.g. variant #2 is AI for libraries NYfBr and NYfLiv.

The AW query interface (called viewAW) has a BioMart [38] style interface, with the exception that the columns can be dynamically selected on the table (see Fig. 2), which eliminates the tedious step of having to reproduce the table to change columns. The interface has many of the details that make querying the database easy, such as maintaining selected columns when a new table is generated. The different viewAW tables are shown on the AW website ‘AW tour’ pages, and a diagram is shown in Figure 1. The transcript and variant queries may be performed on selected sets of libraries, where they can be filtered on user specified coverage and p-value. For example, the user may request to view all transcripts with rank = 1 with at least one missense SNP, and both SNP coverage and read counts AI (p-value<0.01) for both kidney and muscle (results shown in Fig. 2), and then download the corresponding parental transcripts for further analysis in a protein folding program. To support more complex queries, the union, intersection or difference can be computed from two transcript tables. To show the complex architecture of a gene, the AW provides a drawing of the transcripts and variants of a gene, as shown in Fig. 3. It also provides a graphical alignment of parental protein sequences for a specific transcript.

**Figure 4:**
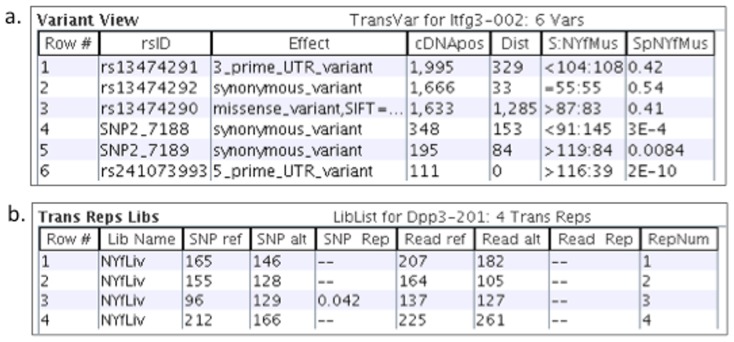
viewAW drilling down into the data. (a) The table shows the variants for an AI transcript. The S:NYfMus column displays the ref:alt SNP coverage for library NYfMus, and the SpNYfMus column shows the corresponding p-values. There are three AI SNPs, where two are ref> alt and the other is alt <ref. (b) The table shows the replicate counts for a transcript. The “SNP Rep” column contains a p-value for any replicate that has significantly different counts from the other replicates.

The various views are necessary to help understand the complexities of the data. There will be inconsistencies, as they are inherently part of genomic data. Fig. 2 shows that just because the transcript is AI, that does not mean that the SNPs are all AI; the #SNPAI column is the number of SNPs that are AI (p-value<0.05) for any library, which is typically less than the #SNPs column. The user can view the SNP p-values and locations using the Draw (Fig. 3) or Variants (Fig. 4a) options. Fig. 4a shows a gene that has two AI SNPs in opposite directions. Fig. 4b shows the consistency of the counts across replicates.

### Transcriptome Computational Workbench (TCW)

Though the TCW has been previously published, it will be briefly summarized here for DE analysis. The TCW takes as input a transcript file and total count files (as provided by the AW pipeline). The runSingleTCW program for building the TCW database includes an interface to edgeR [39], DEGseq [40] and DESeq [41] for computing the differential expression, GOseq [42] for computing the differential expression for gene ontologies, and compares sequences against UniProt [43] using Blast [44]. The TCW query interface also uses the Biomart style and has queries and resulting tables for the transcripts, protein annotations and gene ontologies.

Since there are many GO terms, "GO slim" sets [45] are often used to view the major relevant groups; however, it is difficult to define systematic criteria for selecting these, and predefined sets may be poorly adapted to the project in question. For example, a predefined set may entirely leave out part of the hierarchy that shows DE enrichment in the given experiment. To avoid this problem while still condensing the GO information, the TCW computes a project-specific ‘trimmed’ set, which is designed to highlight non-redundant DE information.

Higher level GO nodes contain lower levels, where a lower level node has either a ‘part-of’ or ‘is-a’ relation to the higher level node. Therefore, there is considerable redundancy in DE enrichment scores between different levels of the hierarchy; hence, the TCW trim algorithm singles out those GOs that appear to be primarily responsible for the enrichment in a given sub-tree using the following rules:

- If a parent (i.e. higher level node) has a better DE score than any of its children, that indicates that the differential effect is happening biologically at the level of the parent, and being inherited by those children, if any, which also are DE-enriched.
- Conversely, if a parent has a worse DE score than one of its children, it indicates that the differential effect is happening biologically at the child level (or below), and being seen in diluted form on the parent.

**Figure 5:**
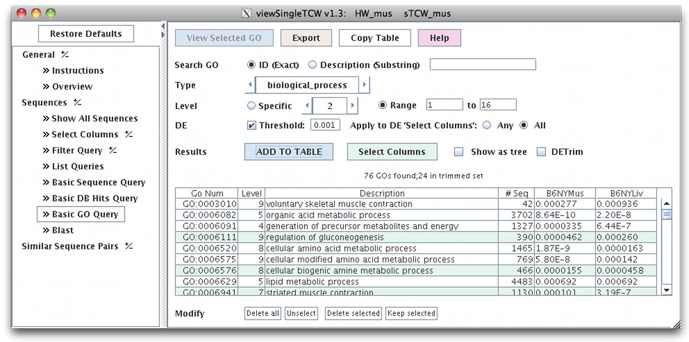
TCW trimmed GO set. All 76 DE-enriched GOs are shown in the table, and the 24 green rows are the trimmed set.

By applying the computation to all GO terms for biological process with a GOseq p-value <0.001 for DE between all B6 reference and hybrid liver and muscle libraries, it reduced 76 terms to 24 (see Fig. 5).

### iPlant Atmosphere Images

Two atmosphere images have been created, one for all the AW software and the other for all TCW software. To run the AW the user only needs to upload their data to their iPlant account and start up an AW instance. The user interacts with the AW software through a desktop emulator. The demo files are part of the image, so the user can immediately try the software. To run the AW software on their own files, they may need to increase their iPlant resource allocations, and then they link to their files on their account and start the pipeline. The user can download the MySQL database to their computer from their iPlant account, download the viewAW executable from the AW website, and make the data and results available to the public for query and display.

### Mouse dataset

Though this manuscript is not intended to provide heterosis mouse results (Goff, in preparation), we used the mouse data from the F1 hybrid progeny in order to demonstrate the AW features and provide some performance results. The dataset has 4 tissues and 4 replicates of Illumina RNA-Seq data (paired end reads, 100 bp); deposited to the Short Read Archive (SRA) at GenBank under accession SRP050309. The hybrid parents are the mouse inbreds (C57BL/6J and BALB/cByJ). The reference genome GRCm38 (strain C57BL/CJ) was downloaded from Ensembl [46]. The variant VCF file was downloaded from the Sanger Centre [47]. Variant effects were obtained from Ensembl Variant Effect Predictor [36].

The processing used the AW-pipeline. Variant calling was only performed for timing since a VCF file was available that was created from genome data. A masked reference genome was created using the VCF file, and trimmed reads were aligned to it and SNP coverage was computed. An AW database was created, which produced the parental transcripts. The trimmed reads were aligned to the transcripts and read counts (reference, alternative and total) were computed and entered into the AW database. The reference protein sequences (output from AW) and the total read counts from both inbreds and the hybrid were entered into a TCW database and annotated with the UniProt [43] mouse taxonomic database. The AW database was used for the analysis in the Results section; the TCW database was only used for Fig. 5.

## Results

The hybrid dataset comprised 15 paired files (one replicate failed). The raw read files were between 7 and 14 gigabytes each, approximately 157 total gigabytes, whereas the final AW database was 400 Mb. Table 2 shows time and memory usage for the different steps, which were performed on an AMD 2.2Ghz system, with 32 CPU and 128 gigabytes of RAM.

**Table 2:** Timing and memory of steps.

Script	Time	Memory	CPUs	Output
QC	2 h	2 G	4	7 M
Trim	2 h	4 G	4	22 G^1^
Align	41 h	4 G	10	38 G
Variants	7 h	1 G	4	50 M
GS Mask	10 m	600 M	1	4 G^2^
SNP coverage	10 m	1 G	4	12 M
Read counts	4 h	3 G	4	300 M
runAW	30 m	2 G	1	400 M

1 Gzip, singletons not saved.

2 Same as the original genome sequence.

The following results are from the AW database overview (shown by selecting the AW Overview tab, see Fig. 2). There were 22,538 genes where 8,785 had at least one SNP and 3,121 had at least one Indel, and 47,234 transcripts where 16,731 had at least one SNP and 4,790 had at least one InDel. Of the 66,529 total SNPs that fall within gene boundaries (excluding introns), 30,432 (46%) were covered by at least 20 reads in at least one library, of which 15,166 (50%) had allele imbalance in at least one library (p-value<0.05). Transitions were the most common SNP substitution type at 72% for CT and its complement GA. There were 232 SNPs and 720 transcripts with a replicate in the opposite direction, and 2,032 transcripts with opposite direction SNPs.

From the overview's variant effect table, the major SNP effects are shown in Table 3. The snpEFF computation provides similar results, but there is some variation (e.g. 7,922 synonymous_variant for #Lib AI). The UTRs had significantly more AI compared to missense (chi-square<0.004), whereas missense SNPs did not have significantly more AI compared to synonymous. The trimming can make a significant difference; using looser parameters produced results where the number of AI missense was greater than the number of AI synonymous SNPs with a p-value <0.0001.

**Table 3:** Allele imbalance of major variant effect categories.

Effect	Count	Covered^1^	AI^2^
3_prime_UTR_variant	32,628	32,288	10,896 (34%)
5_prime_UTR_variant	6,848	4129	1,384(34%)
Missense_variant	10,732	9,647	2,818(29%)
Synonymous_variant	18,998	27,388	7,751(28%)

1 The number of libraries with SNP coverage ≥20 (since there are 4 libraries, the maximum would be 4 x count).

2 The number of libraries with allele imbalance (p-value <0.05); the percent is in relation to the number covered.

After masking the known SNP locations on the B6 reference genome, the SNP coverage counts showed a bias of 51.4%/48.6% in favor of the reference. In comparison, when aligning to an un-masked genome, the bias was 58.5%/41.5%. Even though the residual bias is small in overall terms, it can still result in significant bias in individual detections of AI. Using the viewAW variant query, the total number of AI SNPs with ref>alt for at least one library was 9,226 whereas it was 6,863 for alt>ref (0.74 ratio). Performing the same comparison on transcripts with rank  = 1 using the SNP coverage, there were 2,255 ref>alt and 1,773 alt>ref (0.79 ratio); using the read count, there were 4,392 ref>alt and 3,849 alt>ref (0.88 ratio). Residual reference bias can result from several causes: SNPs that are false-positives (i.e. the alt genome does not actually differ at those loci); SNPs that are uncalled (false-negatives), and hence unmasked, leading to mapping problems at those loci; or indels, which can only be partially addressed by masking even where they are known accurately. These problems may be exacerbated in this dataset since the VCF file was created for alternative genome BALB/cJ, whereas the RNA-Seq data was from the related strain BALB/cByJ.

Of the 47,234 transcripts, 7,843 had rank  = 1. The following queries were performed using filter rank  = 1 so that only one transcript per gene was reported. There is a difference between querying for all transcripts that have at least one SNP with coverage ≥20 (5,575) versus having the summed SNP coverage ≥20 (6,011), where the latter can include transcripts where all SNPs have <20 coverage. There were 2,659 transcripts with SNP AI (p-value <0.01) for at least one library and 5,653 with read AI (p-value <0.01) for at least one library. Using SNP coverage, kidney and liver shared the most AI transcripts with 514, and brain and liver shared the least with 362, whereas with read count, brain and muscle shared the most with 1,732 while again brain and liver shared the least with 1,604.

To select parental transcript sequences for protein folding analysis, there must be at least one missense SNP in order to have any difference in the amino acid sequences. Setting the filters for rank  = 1, at least one missense, and any library with read count p-value <0.01, there were 2,265 transcripts. Since the SNP effects came from Ensembl EVP, they included SIFT annotations, which predicts whether the missense SNP will effect protein function. Adding the filter that at least one SNP must be (SIFT) damaged, there were 451 transcripts.

## Discussion

Publications on both allele specific expression and heterosis include a wide range of analyses. Though the AW and TCW could strive to cover every possible analysis, clever labs will come up with new types of analysis and questions to ask. Moreover, trying to cover every possibility can create confusing software. Therefore, providing the basic analysis allows the user to concentrate on what makes their study unique, i.e. only use custom software on the unique aspects of a study. The following three studies addressed very different problems yet used similar upstream computations of RNA-Seq trimming, determining SNPs, alignment, and performing DE and ASE studies. Bell et al. [48] studied a hybrid crossed from an invasive and native population of the weed *Cirsium.* Pemrumba et al. [49] studied infected and non-infected chickens in two F1 lines, outbred broilers (meat-type) and inbred layer (egg-type), to further their understanding of Marek's disease. Zhia et al. [17] studied heterosis in two stages of roots using the super-rice Xieyou9308 derived from a cross between R9308 (25% japonica) and Xieqingzao B (indica).

As shown in Table 4, these three studies all use the same basic steps until the project specific computations. All three studies require special processing to compute the SNPs, which is the most variable step since the variant calling depends on the attributes of the parents and what genome sequences are available. The following describes what would have to be changed in order for each of these three studies to use the AW and TCW software. Bell et al. would substitute the AW pipeline *Align.pl* in place of using MOSAIK with the 454-contigs. From the AW and TCW graphical interfaces, they could export the contig names and associated values (i.e. AI and DE p-values from AW and TCW respectively) for their project specific analysis. The TCW would at least partially provide their GO analysis. Pemrumba et al. could use the pipeline as is except for the variant calling. The AW and TCW would provide the union queries and variant annotation, but they would need to export information for the DAVID pathway analysis. Zhai et al. could use the pipeline as is except for the variant calling. The AW and TCW would provide their comparison between stages. To compare the DE and AI transcripts, they could export the two tables to further analyze with a custom script. The AW overview provides SNP substitution counts, and the TCW GO analysis would provide much of the GO classification.

**Table 4:** AI and DE processing for three studies.

		Bell et al. [45]	Pemrumba et al. [49]	Zhia et al. [17]
***Input***	*Reference*	454-contigs (invasive)	Chicken genome	Nipponbare rice genome
	*RNA-seq libraries*	Parents, 2 hybrid pools	2 lines, 2 types, 7 replicates	Parents and hybrid, 2 stages, 2 replicates
***Processing***	*Trimming*	Custom script	FASTQC [53], Sickle [54]	Custom script
	*Align*	MOSAIK [55]	Tophat [29]	RSEM [56]
	*Variant Call*	Samtool [11], custom script	Freebayes [57], Merged with VCFtools [14]	Custom script
	*Trans counts*	Assume custom script	HTSeq [58]	
	*Allele Imbalance*	Binomial + FDR	ANOVA	Binomial
	*Differential exp.*	Binomial + FDR	DEseq [41]	EdgeR
***Project specific (for major results only)***	*SNP analysis*	Cis-, trans-acting	Union between types	Comparison between stages, substitutions
	*DE analysis*	Additive, dominance	Union between types	Comparison between stages
	*Other*	GO (TAIR [59], Amigo [60])	ANNOVAR [61], DAVID [62]	Comparison between AI and DE, WEGO [63]

Whether these pipeline substitutions would be adequate is hard to say since Method sections in biology publications rarely cover the analysis to the depth necessary for a user to repeat the analysis; given that they are not software publications, one would not expect that level of detail and justification. However, it is a good reason to use published software when possible, as the software publication should provide the details. Moreover, one must assume that the custom scripts are correct, whereas published software is typically well tested and benefits from feedback from many users. The lack of computational detail also makes it hard to compare results between publications, for example, Zhai et al. identified 480(17%) of 2,793 transcripts with SNP coverage ≥20 to have significant AI (p-value <0.01) in either stage, whereas the AW mouse database shows 2,659 (44%) of the 6,011 transcripts with SNP coverage ≥20 to have significant AI (p-value <0.01) in any library; is this difference real or an artifact of the difference in processing?

Most studies, including the three cited here, do not consider the correlation between ASE SNPs and transcription and translation. A synonymous SNP can have an effect on transcription (reviewed in [50], [51]) and non-synonymous SNP may affect protein folding (reviewed in [52]). Interestingly, in this study, the UTRs had significantly more AI SNPs than the missense SNPs. Though in depth analysis is not provided here, the AW provides the information to further study these phenomena.

## Conclusions

The AW is designed for versatility. By providing the AW pipeline scripts, it provides a ‘bare bones workflow’ for the user, even if the user wants to deviate at any point in the processing. Fortunately, there is now a fair amount of standardization in file formats. Hence, the user can substitute a different program for any of these steps, with the stipulation that they need to adhere to naming conventions. Since there are bound to be questions that a scientist wants to answer which are not in the viewAW, it provides the output of any table in tab delimited format, which the researcher can use for further analysis. This allows the user to have all analysis automated except for the unique questions they are asking. The source code is available, which allows computational biologists to add columns of data or change existing computations. For example, it is very likely that there will be more research on the allele imbalance statistical test, where the Java method for computing the p-values can be altered to experiment with new tests, and then the results can be easily viewed through viewAW.

Though the Methods section on the AW query and display program is short, we consider it the most important part of the AW package. Whereas the AW pipeline will aid scientists with ASE workflow, a person with good bioinformatics skills could install and execute each program, and write scripts for intermediate computations. In contrast, providing the database and versatile query and display requires more sophisticated programming skills, and time that a biological funded project typically does not have. The reasons that a queryable database is so important are: (1) for organized and permanent storage of results, (2) to clearly understand the data, (3) extensibility, and (4) to make it easy to share results with collaborators. It is now typical that data is submitted to Genbank, but each large-scale experiment generates large amounts of results that are only available from the publication, whereas these should also be made publicly available in digital format, preferably via a web-based queryable database. In summary, with the vast amount of high-throughput sequencing results that are being published, it is no longer good practice to have results in flat files and rely on many custom scripts.

The AW code is available from https://code.google.com/p/allele-workbench. The AW and TCW packages (executable files, demo files and documentation) are available from www.agcol.arizona.edu/software (aw and tcw subdirectories, respectively), where each provides a 'tour' of the software. The AW URL has a link to the AW applet to view the hybrid mouse database used in the Results section. The AW and TCW for the full mouse database (48 libraries) are available from www.heterosis.iplantcollaborative.org. The AW and TCW packages are available as Atmosphere Virtual Machine Images at www.iplantcollaborative.org.
